# Impact of an Ivermectin Mass Drug Administration on Scabies Prevalence in a Remote Australian Aboriginal Community

**DOI:** 10.1371/journal.pntd.0004151

**Published:** 2015-10-30

**Authors:** Thérèse M. Kearns, Richard Speare, Allen C. Cheng, James McCarthy, Jonathan R. Carapetis, Deborah C. Holt, Bart J. Currie, Wendy Page, Jennifer Shield, Roslyn Gundjirryirr, Leanne Bundhala, Eddie Mulholland, Mark Chatfield, Ross M. Andrews

**Affiliations:** 1 Menzies School of Health Research, Charles Darwin University, Darwin, Australia; 2 James Cook University, Townsville, Australia; 3 Monash University, Melbourne, Australia; 4 QIMR Berghofer Medical Research Institute, Brisbane, Australia; 5 Telethon Kids Institute, University of Western Australia and Princess Margaret Hospital for Children, Perth, Australia; 6 Miwatj Health Aboriginal Corporation, Nhulunbuy, Australia; 7 La Trobe University, Bendigo, Australia; University of California, San Diego School of Medicine, UNITED STATES

## Abstract

**Background:**

Scabies is endemic in many Aboriginal and Torres Strait Islander communities, with 69% of infants infected in the first year of life. We report the outcomes against scabies of two oral ivermectin mass drug administrations (MDAs) delivered 12 months apart in a remote Australian Aboriginal community.

**Methods:**

Utilizing a before and after study design, we measured scabies prevalence through population census with sequential MDAs at baseline and month 12. Surveys at months 6 and 18 determined disease acquisition and treatment failures. Scabies infestations were diagnosed clinically with additional laboratory investigations for crusted scabies. Non-pregnant participants weighing ≥15 kg were administered a single 200 μg/kg ivermectin dose, repeated after 2–3 weeks if scabies was diagnosed, others followed a standard alternative algorithm.

**Principal Findings:**

We saw >1000 participants at each population census. Scabies prevalence fell from 4% at baseline to 1% at month 6. Prevalence rose to 9% at month 12 amongst the baseline cohort in association with an identified exposure to a presumptive crusted scabies case with a higher prevalence of 14% amongst new entries to the cohort. At month 18, scabies prevalence fell to 2%. Scabies acquisitions six months after each MDA were 1% and 2% whilst treatment failures were 6% and 5% respectively.

**Conclusion:**

Scabies prevalence reduced in the six months after each MDA with a low risk of acquisition (1–2%). However, in a setting where living conditions are conducive to high scabies transmissibility, exposure to presumptive crusted scabies and population mobility, a sustained reduction in prevalence was not achieved.

**Clinical Trial Registration:**

Australian New Zealand Clinical Trial Register (ACTRN—12609000654257).

## Author Summary

Scabies is endemic in many Australian Aboriginal and Torres Strait Islander communities, with 69% of infants infected in the first year of life. Previous mass drug administration (MDA) programs using topical acaricides to decrease scabies prevalence have had varying degrees of success in Australia. We were invited by one community in eastern Arnhem Land to develop and deliver an oral-ivermectin MDA. Utilizing a before and after study design, we measured scabies prevalence through population census with sequential MDAs at baseline and month 12. Scabies prevalence fell from 4% at baseline to 1% at month 6, rising to 9% at month 12 in association with an identified exposure to a presumptive crusted scabies case. For new entries to the cohort at month 12 scabies prevalence was higher at 14%. We were able to demonstrate a reduction in scabies prevalence in the six months after each MDA with a low risk of acquisition (1–2%); however, a sustained reduction was not achieved.

## Introduction

Scabies mites infect up to 300 million people worldwide, most of whom are children living in poverty and overcrowded conditions.[1–3] In remote Australian Aboriginal communities, scabies has been near universal during the first year of life (69%).[4] Secondary infections with highly pathogenic bacterial pathogens *Streptococcus pyogenes* and *Staphylococcus aureus* contribute to high rates of pyoderma in these communities.[5–8] Acute post-streptococcal glomerulonephritis (APSGN) and streptococcal and staphylococcal sepsis,[9],[10] are recognised complications of pyoderma, whereas rheumatic fever, rheumatic heart disease and chronic renal failure are postulated sequelae that all occur in Australian Aboriginal people at the highest rates in the world.[11,12] In contrast, scabies is infrequently seen in non-Indigenous Australians.[2,8,13]

Individuals with scabies classically present with profuse pruritus involving only 5–15 mites per person, whereas an individual with crusted scabies, a rare condition, can have thousands of mites.[14,15] Well documented to occur in immune compromised hosts, most Aboriginal people identified with crusted scabies have no definable immune defect.[16] People with crusted scabies are highly infectious and have been identified as core transmitters in scabies epidemic cycles and institutional outbreaks.[3,16,17] Prior to 1996 and the introduction of ivermectin in Northern Territory (NT) Australia, there was a 5-year mortality rate of up to 50% for people with crusted scabies.[16]

Mass drug administration (MDA) programs using topical acaricides to decrease scabies prevalence have had varying degrees of success in Australia.[5,8,13] Due to high endemicity, high transmissibility of infestations, low treatment uptake and limited regional coverage, the presence of crusted scabies in communities and mobility of regional populations, a sustained reduction in prevalence has not been achieved to date in remote Aboriginal communities.[1,8,18] Having an established collaboration through the East Arnhem Healthy Skin Program [1,19,20] which demonstrated poor uptake of topical acaricides in household contacts,[1] we were invited by one community in eastern Arnhem Land to develop a proposal for an oral-ivermectin MDA targeting both scabies and strongyloidiasis. Strongyloidiasis is an infection with the intestinal nematode parasite, *Strongyloides stercoralis*, for which ivermectin is the first-line treatment.[21] Here we report the outcomes against scabies of the MDA program designed in collaboration with the participating community.

## Methods

The setting was a remote island community, 550km from Darwin, Australia with an estimated population of 2121.[22] Most residents lived in the main community; 200–400 lived in one of 10 associated homelands outside the community (five of which were accessible only by air/water).

In consultation with the community, we designed a staged roll-out of two MDAs, implemented 12 months apart for the respective households/homelands. MDAs are typically designed to be implemented within a short time frame to maximise reduction of infective stages. However, our consultations with the community stressed the need for a more extended roll-out period to encompass house to house consultation, screening and treatment involving locally trained workers. There were 159 houses in the main community at the start of the project and 165 houses at the second MDA. The program was evaluated in a before and after study design.

We conducted population censuses in 2010 (baseline) and 2011 (month 12) to screen for scabies and strongyloidiasis that all residents were eligible to participate in. The MDA was delivered at the same time using an allocated drug regimen (Table 1). Two surveys were conducted six months after each MDA (month 6 and 18) to: a) follow-up participants who were positive for scabies and/or had an equivocal/positive *Strongyloides* result in the census six months prior, b) screen a computer-generated random sample of participants who were negative for both scabies and strongyloidiasis in the census six months prior and c) follow-up contacts of scabies acquisitions diagnosed at month 6 or 18. Given the staged program roll-out, subsequent visits to households were scheduled to accommodate the planned 6–12 month follow-up timeline as per the study protocol [23].

**Table 1:** Drug regimen for MDAs and treatment of scabies.

Group	Medications administered at baseline & month 12	Treatment after 10–42 days for those diagnosed with scabies
**Weight**	**Day 1–3**	**Day 10–42**
**<3.5 kg**	Topical 10% crotamiton daily for 3 consecutive days	Topical 10% crotamiton daily for 3 consecutive days
**3.5 kg<6 kg**	Topical 5% permethrin	Topical 5% permethrin
**6 kg <15 kg**	Topical 5% permethrin & oral albendazole 200 mg (6–10 kg) or 400 mg (10-<15 kg) daily for 3 consecutive days	Topical 5% permethrin
**Not pregnant and weight ≥15 kg**	Oral ivermectin 200 μg/kg	Oral ivermectin 200 μg/kg
**Pregnant**	Topical 5% permethrin	Topical 5% permethrin

An allocated drug regimen for both scabies and strongyloidiasis was delivered based on weight and pregnancy status (Table 1). All non-pregnant participants who weighed ≥15 kg were administered a single dose of ivermectin 200 μg/kg at baseline and at month 12. Those ineligible for ivermectin received either topical 5% permethrin or 10% crotamiton. Treatment was repeated after 2–3 weeks if scabies and/or strongyloidiasis were diagnosed. All household contacts of participants diagnosed with scabies were either treated as part of the MDA or referred to the clinic. At the month 6 and 18 surveys, those diagnosed with scabies and their household contacts were provided with treatment and follow-up. Strongyloidiasis cases were treated but not their family contacts.

Residents were excluded from the MDA if they had an allergy to any components of the allocated drug regimen or had received the eligible study medication in the previous seven days. All female study participants aged 12–45 years had the option of a urinary test to determine pregnancy status as ivermectin safety in pregnancy has not been established.[24] Those not tested were allocated to the same treatment regimen as pregnant women. Pregnancy testing and medication administration was undertaken in portable work stations ensuring individual privacy. Adherence with the allocated drug regimen was monitored by direct observation of oral therapy and through verbal discussions with those applying topical acaricides.

Scabies was diagnosed clinically from observation of exposed skin. We classified scabies as: scabies-like lesions in a person who had either an itch, lesions in a typical location, or a household member with an itch. We accepted typical scabies lesions as being burrows, erythematous papules and macules, scales, vesicles, bullae, crusts, pustules, nodules and/or excoriations located in the finger web spaces, flexor surfaces of the wrists and elbows, axillae, head, feet, palms or buttocks in children or male genitalia and female breasts where assessed. Flipcharts [25] were used by Aboriginal Health Practitioners, Registered Nurses and ACWs to assist with the diagnosis of scabies and pyoderma. Participants who had a clinical diagnosis of suspected crusted scabies were referred to the local health service for laboratory confirmation and medical care according to locally developed guidelines which have been adopted internationally.[26]

Data were analysed using Stata 13 (StataCorp LP). Scabies prevalence at baseline and month 12 was calculated as a proportion of those seen who were diagnosed with scabies. At month 6 and 18 surveys, prevalence was determined as a weighted average of (i) treatment failure rate—the prevalence for participants seen with scabies at the survey who had scabies at the population census six months prior, and (ii) acquisition rate—the prevalence for participants seen at the survey who did not have scabies at the census six months prior. In determination of scabies acquisition, we also included those who were *Strongyloides* positive/equivocal but scabies negative in the denominator along with the computer generated randomly selected negatives from six months prior, as there was no relation between scabies and strongyloidiasis at baseline or month 12. Pyoderma prevalence was reported at baseline and month 12. Per protocol treatment was calculated as a proportion of those eligible for the drug regimen who were administered medication as outlined in Table 1.

Data entry was validated by double entering 15% of the records. The data entry error rate for variables used in the analysis was <5%. Data is available from the Dryad Digital Repository. [27]

### Project Registration and Ethics Statement

The project was registered with the Australian New Zealand Clinical Trial Register (ACTRN—12609000654257)[23] and received ethical approval from Human Research Ethics Committee of the Northern Territory Department of Health and Menzies School of Health Research (EC00153—project 09/34).

Study recruitment was conducted by Aboriginal Community Workers (ACWs) who had completed a nationally accredited training program (Certificate II in Child Health Research 70131NT). The ACWs visited each house to discuss the project with family members and establish a household occupancy list. Ascertainment of written informed consent was obtained using a pictorial flipchart that incorporated a culturally-appropriate process to explain the project.[28] Parents or registered caregivers provided written consent for children aged <18 years and additional written assent was obtained from children aged 12-<18 years.

## Results

At baseline, there were 1256 residents on the household occupancy lists in the population census (March-September 2010), of which 1013 (81%) consented to participate. Most participants (n = 960, 95%) were seen over a four month period (April-July). The median number of participants per house was nine (IQR 4–13) from 127 (80%) houses visited. Non-participating households were mostly those occupied by non-Aboriginal residents working in the community. Seven of the 10 homelands consented to participate; one refused whilst residents from the other two homelands were seen in houses in the main community. A total of 1002 participants had data recorded on scabies at baseline, with scabies data missing for the remaining 11 participants (1%).

At month 12, there were 1163 residents on the household occupancy lists in the second population census (April-October 2011), of whom 1060 (91%) participated (~150 per month). There were 700 (66%) whom had also been seen at baseline and 360 (34%) new participants not previously seen. The median number of participants per house was 8 (IQR 3–12) from 133 (81%) houses visited. The median time per person between the baseline and month 12 census was 14 months (IQR 12–17 months). Most participants (96%) received the per protocol MDA regimen at baseline and 12, whilst 72% of those diagnosed with scabies received their second treatment as per protocol. No adverse events following administration of medications were reported.

### Scabies Prevalence

**Fig 1:**
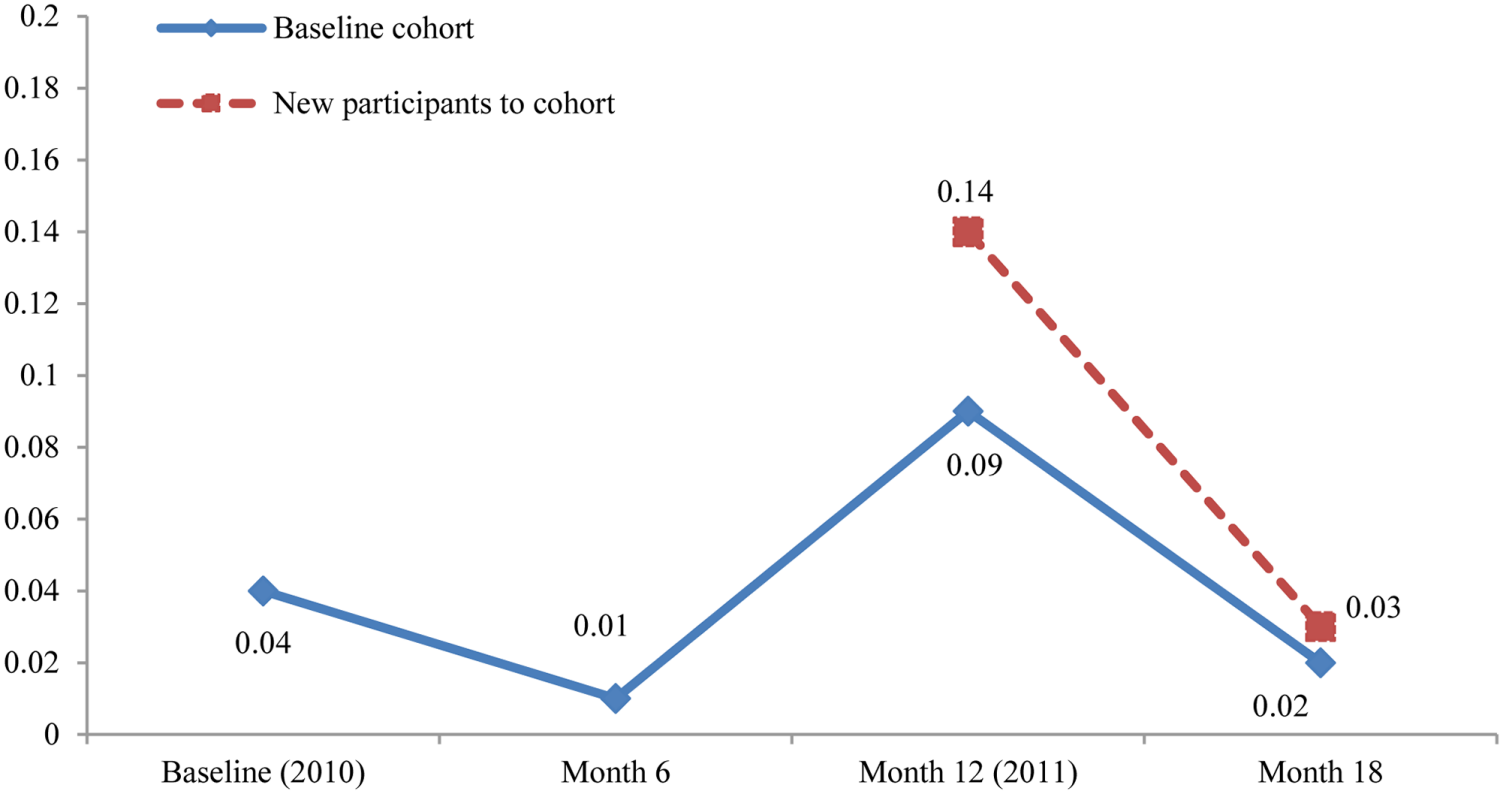
Scabies prevalence at population censuses (2010 & 2011) and month 6 & 18 surveys.

Scabies prevalence among the baseline cohort was 4% and remained relatively stable during the initial assessment period (2%, 6%, 3% and 5% per month from April-July 2010 respectively when 91% of the baseline cohort were seen). At the month 6 survey, prevalence was 1% but increased to 9% at month 12 (5% absolute increase from baseline to month 12 for the baseline cohort) (Fig 1). At month 18, prevalence fell to 2%. The median age of participants with scabies was 11 years (IQR 6–38 years) with more females at baseline diagnosed with scabies than males (Table 2). Of the 42 participants diagnosed with scabies, 8/35 (23%) had infected scabies.

**Table 2:** Participant details for the population census at month 0 and 12.

	Yes scabies	No scabies	Total
***Month 0***	***n = 42***	***n = 960***	***n = 1002***
Median age (IQR)	11 (6–40)	21 (9–37)	21 (9–37)
***Gender***			
Male	9 (21%)	484 (50%)	493 (49%)
Female	33 (79%)	476 (50%)	509 (51%)
***Month 12***	***n = 113***	***n = 947***	***n = 1060***
Median age (IQR)	11 (6–19)	22 (10–36)	21 (9–35)
***Gender***			
Male	59 (52%)	478 (50%)	537 (51%)
Female	54 (48%)	469 (50%)	523 (49%)

Prevalence among the baseline cohort had increased from 4% to 9% at month 12, whereas prevalence among new entries to the cohort (those seen for the first time at month 12) was 14% (Fig 1). In addition to the new cohort entries, the increased prevalence at month 12 was influenced by a cluster of cases epidemiologically linked to a participant diagnosed with presumptive crusted scabies. Prevalence within the baseline cohort of those who were known contacts rose from 7% (7/96) at baseline to 18% (17/96) at month 12, whereas prevalence amongst others who were not known contacts within the baseline cohort rose from 4% (23/598) at baseline to 8% (46/604) at month 12. Of the 113 participants diagnosed with scabies, 34/105 (32%) had infected scabies.

The presumptive crusted scabies case was identified in May 2011, a school age participant who had been receiving topical acaricide treatment from the school nurse every two weeks for the previous two months. With support from nine public health personnel who joined the study team, we identified 13 priority houses for follow-up, three of which were houses where the presumptive crusted scabies participant had been living over the previous four weeks, and 10 other households that had school contacts with scabies.

**Fig 2:**
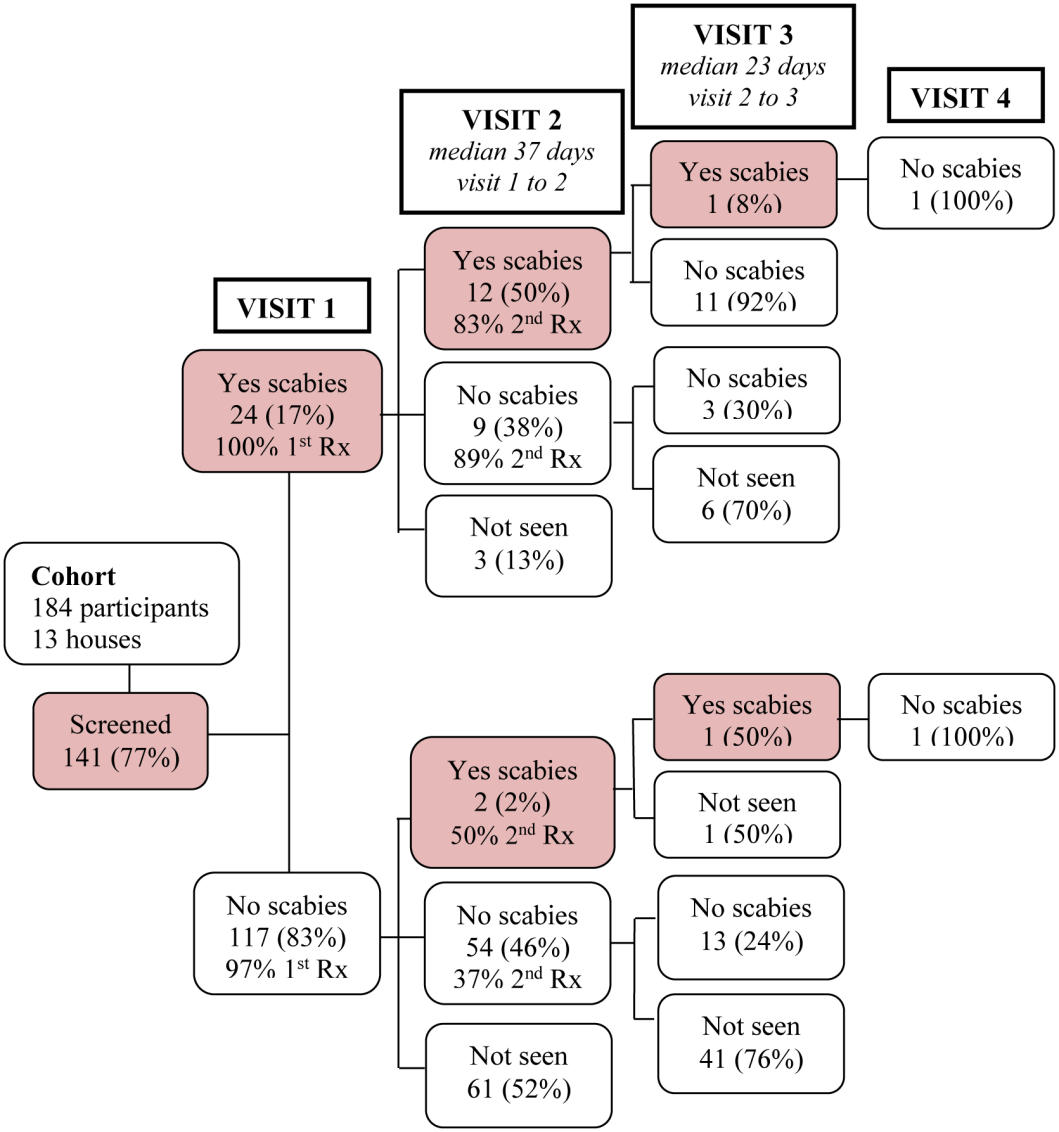
Flowchart of visits to participants in 13 priority houses.

There were 184 people identified as residing in these 13 houses of whom 141 (77%) were seen; a median of 13 (IQR 10–18) participants per house (Fig 2). Of the 141 participants seen, 91 (65%) were from the baseline cohort of whom 16 (18%) had scabies at month 12. Of the 50 new participants seen for the first time in the priority houses, eight (16%) had scabies. Scabies prevalence within these 13 households collectively was 17% (n = 24). Almost all (98%) of those seen received ivermectin or 5% permethrin at the first visit.

On follow-up, 77/184 residents (42%) from the 13 priority houses were seen again at visit 2, median 37 days (IQR 23–42) after visit 1, with an acquisition rate of 4%. Of the 24 participants observed with scabies lesions at visit 1, 18 (75%) were re-treated at visit 2 (12 had lesions present when reviewed). Follow-up of these priority houses was completed within two months.

**Fig 3:**
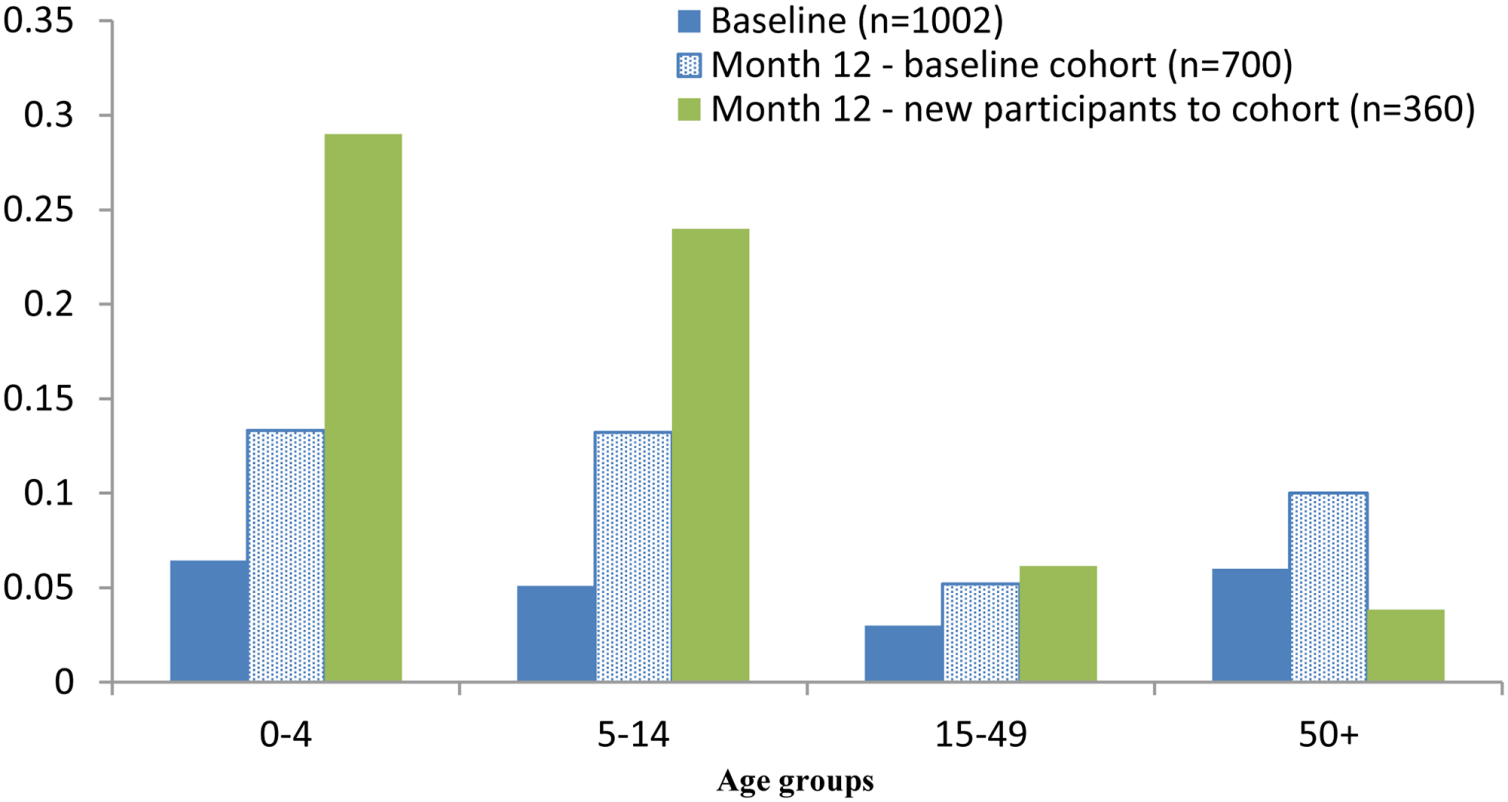
Scabies prevalence at baseline & month 12, by age group.

**Fig 4:**
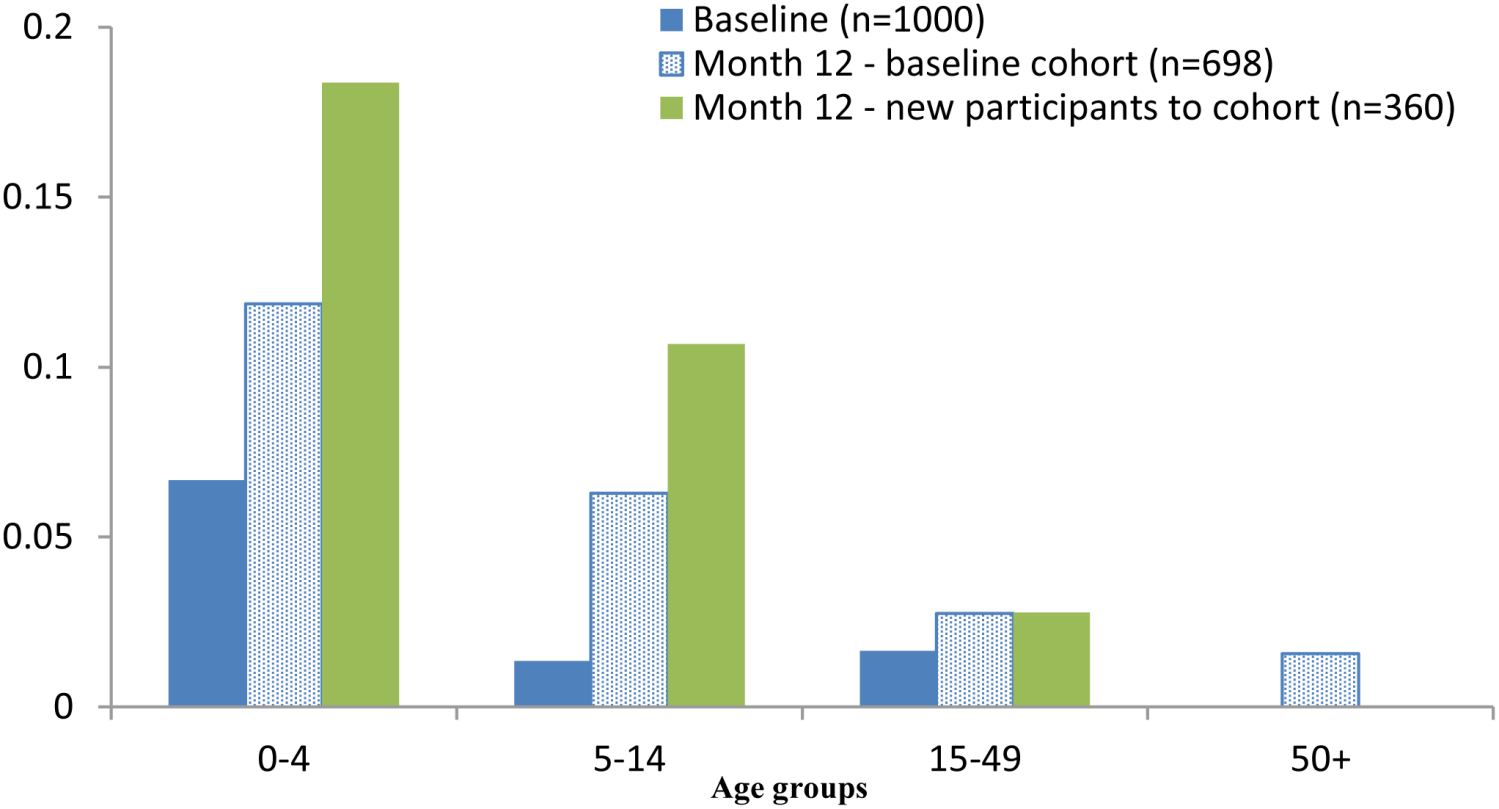
Pyoderma prevalence at baseline & month 12, by age group.

The increase in scabies prevalence at month 12 was most evident among children <15 years of age and was highest amongst new entries to the cohort (Fig 3). Pyoderma prevalence where the sores were described as purulent or crusted also increased amongst these age groups at month 12 (Fig 4).

### Month 6 and 18

Scabies treatment failures and acquisition were low throughout the study period (S1 and S2 Tables). The treatment failure rate was 6% (2/35) at month 6 and 5% (5/91) at month 18. The acquisition rate was 1% (4/352) at month 6 and 2% (6/276) at month 18. The median time between participant visits from baseline to month 6 was six months (IQR 5–7 months) and from month 12 to 18, eight months (IQR 7–10).

## Discussion

In our study, MDA incorporating ivermectin had a demonstrable but relatively short-term impact on scabies prevalence. In the six-months following each MDA, both the low overall prevalence (1–3%) and the low acquisition rates (1–2%) suggest that transmission was substantially reduced. However, the rapid rise in prevalence at month 12 highlights that an MDA program, where utilised, needs to be incorporated with a multi-faceted control program and ongoing surveillance in the community.

MDAs have been used to control and eliminate diseases for more than 25 years.[29] Ivermectin is one of the most commonly used drugs worldwide in the treatment of strongyloidiasis, lymphatic filariasis, and onchocerciasis.[30] It is increasingly being used to treat other parasitic infections including scabies,[31] pediculosis capitis [32] and malaria.[33] In 2014, Merck Sharp and Dhome updated the indications for ivermectin use to include treatment of crusted scabies and classical scabies if topical treatment is ineffective.[24,34].

Scabies is a neglected tropical disease,[35] ubiquitous in Australian Aboriginal and Torres Strait Islander communities, despite repeated MDAs with topical acaricides.[8],[3] Infestations are highly transmissible,[3] and as this study shows, prevalence escalates in the presence of high exposure (prevalence amongst known contacts of the presumptive crusted scabies case rose from 7% at baseline to 18% at month 12) and a high proportion of mobility (36% new entries to the cohort at month 12, of whom 14% had scabies). Others have shown the impact of exposure to crusted scabies [36] and overcrowded living conditions [1] on scabies prevalence. Outbreaks [37] and high scabies prevalence [38] have previously been linked with epidemics of APSGN, the sequelae of a post streptococcal infection that is common in developing countries and Indigenous populations.[10]

The participation rate among residents within the community was noteworthy (80–95%) encompassing an informed consent process implemented with and by the community. Under the guidance of elders and key community stakeholders, the development of a pictorial flipchart that incorporated a culturally-appropriate process to explain the project was fundamental in obtaining informed consent.[28] The flipchart incorporated a local story well known in the community which we had gained specific approval to use and translate into local language. That some members declined participation is testament to the culturally appropriate processes enabled within this study. Moreover, the process of ongoing engagement and the culturally acceptable arrangements regarding pregnancy testing, screening and steady (as opposed to rapid) roll-out of the program were integral to the reach achieved over the course of the study.

A previous attempt to implement an ivermectin MDA for scabies control in Queensland Aboriginal communities in the early 1990s did not proceed due to administrative concerns about medication safety and informed consent.[39] Instead the team conducted a MDA with ivermectin in the Solomon Islands and showed ivermectin to be safe and effective with low scabies prevalence persisting for at least 32 months.[31] The longer-term duration of benefit however, is unclear as there was no ongoing active surveillance. In Fiji, no significant difference was found between MDAs with either ivermectin or benzyl benzoate after 24–28 days.[40]

To date, the use of ivermectin to treat scabies has not been associated with any serious adverse effects nor were any observed in our study. However, it is recommended that ivermectin not be administered to pregnant women or children who are younger than five years of age or in those who weigh less than 15 kg. This recommendation is due to theoretical concerns regarding potential neurotoxicity and a lack of safety data. Although there have been no reports of foetal problems when ivermectin has been administered in pregnancy to thousands of women, caution is still recommended.[41] While the safety of ivermectin at the extremes of age remains to be conclusively established, there is increasing evidence suggesting that the use of ivermectin in children <5 years is safe.[26]

The high proportion of new entries to the cohort at the month 12 census (36%) coincided with a large funeral that was attended by visitors from other communities who were camping in tents in the house yards of relatives. At this time, many local residents were also displaced from their homes into tents or other people’s homes as their houses were being refurbished or demolished and rebuilt, as part of a government initiative to address housing shortages in Aboriginal communities.[42] This change in population dynamics is considered highly mobile by Australian mainstream standards, but does not reflect the stability reflected by the customary attachment of Aboriginal people to their home community and the regional area.[43]

The increased scabies prevalence at month 12 was notable in the 0–14 year age group and in particular for those new participants to the cohort. Young children are particularly susceptible to scabies infestations [2,4,44] and, as shown in this study, are more likely than adults to show a change in prevalence. For population surveillance of scabies it has previously been recommended that this is best achieved by monitoring the prevalence in young children,[4] a recommendation that is further supported by this study.

At baseline there was concern about inter-observer variation in the diagnosis of scabies as more females (n = 33) than males (n = 9) had been diagnosed with scabies. These concerns were dispelled after reviewing the names of the researchers screening the children (for whom the majority of scabies were diagnosed) and found that the female researchers, who at that time had more experience in diagnosing scabies than the male researchers, had been conducting most of the skin checks for male and female children. Thereafter we conducted regular reviews of screening processes in the field and from photographs taken, to improve consistency in diagnosis and reduce inter observer variation.

It was also apparent to our community-based research team, that the relationship built over the course of the team’s work meant that by month 12 it was relatively commonplace for households to seek out the research team to assist in making their homes scabies free, and to send family members who had not been present on the day the family were seen to the research office for screening and treatment. We acknowledge that this may have introduced a screening bias in the latter part of the study but the increased scabies prevalence at month 12 amongst those who had been seen at baseline indicates that the increase in prevalence was not an artefact of care-seeking behaviour.

The rise in scabies prevalence at month 12 coincided with: a cluster of cases epidemiologically-linked to an individual with presumptive crusted scabies, a high prevalence amongst new entries to the cohort (an indicator of the impact of high population mobility), and an increased prevalence amongst members of the baseline cohort who did not have a known exposure to the suspected crusted scabies case (4% to 8%). This demonstrated how readily scabies prevalence can increase. Control measures were able to be implemented promptly as the research team had commenced the second house to house population census and MDA and were able to coordinate the response with the local PHC services and community. Of note, was an outbreak of APSGN [45] occurring at the same time in another large NT community that public health personnel were responding to. Scabies prevalence in this community for children aged 1–17 years was 3% (n = 8) and 40.5% (n = 219) for purulent or crusted sores. Personal communication from the public health unit revealed there had been three cases of ARF and no cases of APSGN reported in the region in the four months following the outbreak.

Our study provides evidence that ivermectin based MDAs can have a role in reducing scabies prevalence but also highlights that maintaining a reduction requires ongoing surveillance,[4] diagnosis and chronic case management of individuals with crusted scabies,[18,34] and ongoing engagement with community members that has a particular focus on households and close contacts.[1] Due to the customary movements of Aboriginal people, regional approaches to decrease re-introduction of scabies from neighbouring communities needs to be considered.

## Supporting Information

S1 Table Scabies at month 6 / participants seen at month 6 [participants seen at month 0], by scabies status and *Strongyloides* status at month 0. (DOCX)

S2 Table Scabies at month 18 / participants seen at month 18 [participants seen at month 12], by scabies status and *Strongyloides* status at month 12. (DOCX)

S1 Checklist STROBE Checklist. (DOC)
