# Supplementary material for: Impact of an Ivermectin Mass Drug Administration on Scabies Prevalence in a Remote Australian Aboriginal Community
Source: PLoS Negl Trop Dis. 2015 Oct 30;9(10):e0004151. doi: 10.1371/journal.pntd.0004151 (PMC4627839; doi:10.1371/journal.pntd.0004151)
Supplement: S1 Table — (DOCX) [file pntd.0004151.s001.docx]

**Supplementary Data**

The numbers in red are participants seen at the month 6 and 18 surveys who had also been seen at the population census six months prior. The numbers in black brackets [..] are the participants seen in each category from the population census six months prior. The red denominator in the second column and second row (*Strongyloides* negative*,* Scabies absent) are participants that were seen from a list of 200 randomly selected negatives. The figures in black brackets [..] in the third row (*Strongyloides* equivocal/positive) and third column (Scabies present) are those that were to be followed up at the month 6 and 18 surveys. Not all participants that were to be followed up were able to be located for review at the month 6 and 18 surveys.

**Table S1. Scabies at month 6 / participants seen at month 6 [participants seen at month 0], by scabies status and *Strongyloides* status at month 0.**

|  | Scabies absent  baseline | Scabies present  baseline | Scabies unknown baseline | Total |
| --- | --- | --- | --- | --- |
| *Strongyloides* negative baseline | 2/141 (1.4%)  [528] | 2/19 (10.5%)  [22] | 0/0  [ 9] | 4/160 (2.5%)  559 |
| *Strongyloides* equivocal/positive  baseline | 2/211 (0.9%)  [285] | 0/11  [14] | 0/1  [ 1] | 2/223 (0.9%)  300 |
| *Strongyloides* unknown  baseline | 0/0  [147] | 0/5  [6] | 0/0  [ 1] | 0/5  154 |
| Total | 4/352 (1.1%)  [960] | 2/35 (5.7%)  [42] | 0/1  [11] | 6/388 (1.5%)  [1,013] |

*Note. 2/7 extra household contacts were also examined at month 6 (not included in the above), and found positive for scabies. Scabies status was never unknown at month 6 for those seen.*

- Prevalence baseline: 42/1002 = 4% (11 participants had missing data for scabies)
- Treatment failures at month 6: 2/35 (6%) with scabies at baseline who still had scabies at month 6
- Scabies acquisition at month 6: 4/352 (1%) with no scabies at baseline who had acquired scabies at month 6
- Prevalence month 6: (2/35)*42 + (4/352)*960]/1002 = 13/1002 = 1%
