# Supplementary material for: Impact of an Ivermectin Mass Drug Administration on Scabies Prevalence in a Remote Australian Aboriginal Community
Source: PLoS Negl Trop Dis. 2015 Oct 30;9(10):e0004151. doi: 10.1371/journal.pntd.0004151 (PMC4627839; doi:10.1371/journal.pntd.0004151)
Supplement: S2 Table — (DOCX) [file pntd.0004151.s002.docx]

**Supplementary Data**

The numbers in red are participants seen at the month 6 and 18 surveys who had also been seen at the population census six months prior. The numbers in black brackets [..] are the participants seen in each category from the population census six months prior. The red denominator in the second column and second row (*Strongyloides* negative*,* Scabies absent) are participants that were seen from a list of 200 randomly selected negatives. The figures in black brackets [..] in the third row (*Strongyloides* equivocal/positive) and third column (Scabies present) are those that were to be followed up at the month 6 and 18 surveys. Not all participants that were to be followed up were able to be located for review at the month 6 and 18 surveys.

**Table S2. Scabies at month 18 / participants seen at month 18 [participants seen at month 12], by scabies status and *Strongyloides* status at month 12.**

|  | Scabies absent  Month 12 | Scabies present  Month 12 | Total |
| --- | --- | --- | --- |
| *Strongyloides* negative  Month 12 | 4/132 (3%)  [675] | 2/55 (3.6%)  [65] | 6/187 (3.2%)  740 |
| *Strongyloides*  equivocal/positive  Month 12 | 2/144 (1.4%)  [189] | 3/19 (15.7%)  [25] | 5/163 (3%)  214 |
| *Strongyloides* unknown  Month 12 | 0/0  [83] | 0/17  [23] | 0/17  106 |
| Total | 6/276 (2.1%)  [947] | 5/91 (5.4%)  [113] | 11/367 (2.9%)  [1,060] |

*Note. 4/14 extra household contacts were also examined at month 18 (not included in the above), and found positive for scabies. Scabies status was unknown at month 18 for one person – not included in above table.*

- Prevalence month 12: 113/1060 = 11%
- Treatment failures at month18: 5/91 (5%) with scabies at month 12 still had scabies at month 18
- Scabies acquisition month 18: 6/276 (2%) with no scabies at month 12 had acquired scabies at month 18
- Prevalence month 18: [(5/91)*113 + (6/276)*947]/1060 = 27/1060 = 3%
