# Supplementary material for: Impact of an Ivermectin Mass Drug Administration on Scabies Prevalence in a Remote Australian Aboriginal Community
Source: PLoS Negl Trop Dis. 2015 Oct 30;9(10):e0004151. doi: 10.1371/journal.pntd.0004151 (PMC4627839; doi:10.1371/journal.pntd.0004151)
Supplement: S1 Checklist — (DOC) [file pntd.0004151.s003.doc]

STROBE Statement—checklist of items that should be included in reports of observational studies

|  | Item No | Recommendation |
| --- | --- | --- |
| **Title and abstract** | 1 | (*a*) Indicate the study’s design with a commonly used term in the title or the abstract  Abstract – Methods “*Utilizing a before and after study design, we measured scabies prevalence through population census with sequential MDAs at baseline and month 12.”* p. 1 |
|  |  | (*b*) Provide in the abstract an informative and balanced summary of what was done and what was found. p. 1 & 2.  ***Methods***  *Utilizing a before and after study design, we measured scabies prevalence through population census with sequential MDAs at baseline and month 12. Surveys at months 6 and 18 determined disease acquisition and treatment failures. Scabies infections were diagnosed clinically with additional laboratory investigations for crusted scabies. Non-pregnant participants weighing ≥15 kg were administered a single 200 μg/kg ivermectin dose, repeated after 2-3 weeks if scabies was diagnosed, others followed a standard alternative algorithm.*  ***Principal findings***  *We saw >1000 participants at each population census. Scabies prevalence fell from 4% at baseline to 1% at month 6. Prevalence rose to 9% at month 12 amongst the baseline cohort in association with an identified exposure to a presumptive crusted scabies case with a higher prevalence of 15% amongst new entries to the cohort. At month 18, scabies prevalence fell to 2%. Scabies acquisitions six months after each MDA were 1% and 2% whilst treatment failures were 6% and 5% respectively.* |
| Introduction – p. 3. | | |
| Background/rationale | 2 | Explain the scientific background and rationale for the investigation being reported, p. 3, ‘*Mass drug administration (MDA) programs using topical acaricides to decrease scabies prevalence have had varying degrees of success in Australia. ……………’* |
| Objectives | 3 | State specific objectives, including any prespecified hypotheses, p. 3, *‘Here we report the outcomes against scabies of the MDA program designed in collaboration with the participating community.’* |
| Methods – p. 4-6. | | |
| Study design | 4 | Present key elements of study design early in the paper, p. 4, *‘In consultation with the community, we designed a staged roll-out of two MDAs, implemented 12 months apart for the respective households/homelands. ……………………..’* |
| Setting | 5 | Describe the setting, locations, and relevant dates, including periods of recruitment, exposure, follow-up, and data collection, p. 4-5. *‘The setting was a remote island community, 550km from Darwin, Australia with an estimated population of 2121. … We conducted population censuses in 2010 (baseline) and 2011 (month 12) to screen for scabies and strongyloidiasis that all residents were eligible to participate in. The MDA was delivered at the same time using an allocated drug regimen (Table 1).…..’* |
| Participants | 6 | 1. *Cohort study*—Give the eligibility criteria, and the sources and methods of selection of participants. Describe methods of follow-up, p. 4-5, *‘An allocated drug regimen for both scabies and strongyloidiasis was delivered based on weight and pregnancy status (Table 1). All non-pregnant participants who weighed ≥15 kg were administered a single dose of ivermectin 200 μg/kg at baseline and at month 12. Those ineligible for ivermectin received either topical 5% permethrin or 10% crotamiton. Treatment was repeated after 2-3 weeks if scabies and/or strongyloidiasis were diagnosed. All household contacts of participants diagnosed with scabies were either treated as part of the MDA or referred to the clinic. ‘* 2. *Cross-sectional study*—Give the eligibility criteria, and the sources and methods of selection of participants, p. 5*, ‘At the month 6 and 18 surveys, those diagnosed with scabies and their household contacts were provided with treatment and follow-up. Strongyloidiasis cases were treated but not their family contacts.’* |
| Variables | 7 | Clearly define all outcomes, exposures, predictors, potential confounders, and effect modifiers. Give diagnostic criteria, if applicable, p. 5-6, *‘Scabies was diagnosed clinically from observation of exposed skin. We classified scabies as: scabies-like lesions in a person who had either an itch, lesions in a typical location, or a household member with an itch ……………….’* |
| Data sources/ measurement | 8* | For each variable of interest, give sources of data and details of methods of assessment (measurement). Describe comparability of assessment methods if there is more than one group, p. 5-6, ‘*We accepted typical scabies lesions as being burrows, erythematous papules and macules, scales, vesicles, bullae, crusts, pustules, nodules and/or excoriations located in the finger web spaces, flexor surfaces of the wrists and elbows, axillae, head, feet, palms or buttocks in children or male genitalia and female breasts where assessed. Flipcharts were used to assist with the diagnosis of scabies and pyoderma, which were made by Aboriginal Health Practitioners, Registered Nurses and ACWs.’* |
| Bias | 9 | Describe any efforts to address potential sources of bias, p. 4, ‘*Study recruitment was conducted by Aboriginal Community Workers (ACWs) who had completed a nationally accredited training program (Certificate II in Child Health Research 70131NT). The ACWs visited each house to discuss the project with family members and establish a household occupancy list.’* |
| Study size | 10 | Explain how the study size was arrived at, p. 4, *‘We conducted population censuses in 2010 (baseline) and 2011 (month 12) to screen for scabies and strongyloidiasis that all residents were eligible to participate in……………. Two surveys were conducted six months after each MDA (month 6 and 18) to: a) follow-up participants who were positive for scabies and/or had an equivocal/positive Strongyloides result in the census six months prior, b) screen a computer-generated random sample of participants who were negative for both scabies and strongyloidiasis in the census six months prior and c) follow-up contacts of scabies acquisitions diagnosed at month 6 or 18.’* |
| Quantitative variables | 11 | Explain how quantitative variables were handled in the analyses. If applicable, describe which groupings were chosen and why, p. 6. *‘Scabies prevalence at baseline and month 12 was calculated as a proportion of those seen who were diagnosed with scabies. At month 6 and 18 surveys, prevalence was determined as a weighted average of (i) treatment failure rate - the prevalence for participants seen with scabies at the survey who had scabies at the population census six months prior, and (ii) acquisition rate - the prevalence for participants seen at the survey who did not have scabies at the census six months prior. In determination of scabies acquisition, we also included those who were Strongyloides positive/equivocal but scabies negative in the denominator along with the computer generated randomly selected negatives from six months prior, as there was no relation between scabies and strongyloidiasis at baseline or month 12.’* |
| Statistical methods | 12 | (*a*) Describe all statistical methods, including those used to control for confounding, as above in 11. |
|  |  | (*b*) Describe any methods used to examine subgroups and interactions, as above in 11. |
|  |  | (*c*) Explain how missing data were addressed, as above in 11. |
|  |  | (*d*) *Cohort study*—If applicable, explain how loss to follow-up was addressed, as above in 11. *Cross-sectional study*—If applicable, describe analytical methods taking account of sampling strategy, as above in 11. |

Continued on next page

| Results p. 6-11 | | |
| --- | --- | --- |
| Participants | 13* | (a) Report numbers of individuals at each stage of study—eg numbers potentially eligible, examined for eligibility, confirmed eligible, included in the study, completing follow-up, and analysed, p. 6 & 7, *‘At baseline, there were 1251 residents on the household occupancy lists in the population census (March-September 2010), of which 1013 (81%) consented to participate. Most participants (n=910, 90%) were seen over a four month period (April-July). The median number of participants per house was 13 (IQR 9-18) from 127 (80%) houses visited.’* |
|  |  | (b) Give reasons for non-participation at each stage, p. 6 & 7, ‘*Non-participating households were mostly those occupied by non-Aboriginal residents working in the community. Seven of the 10 homelands consented to participate; one refused whilst residents from the other two homelands were seen in houses in the main community. A total of 1002 participants had data recorded on scabies at baseline, with scabies data missing for the remaining 11 participants (1%)…………….’* |
| Descriptive data | 14* | (a) Give characteristics of study participants (eg demographic, clinical, social) and information on exposures and potential confounders, p. 7, *‘Table 2. Participant details for the population census at month 0 and 12.’* |
|  |  | (b) Indicate number of participants with missing data for each variable of interest, Supplementary data,*’****Table S1. Scabies at month 6 / participants seen at month 6 [participants seen at month 0], by scabies status and Strongyloides status at month 0. Table S2………………..’*** |
|  |  | (c) *Cohort study*—Summarise follow-up time (eg, average and total amount), p. 6-7, *‘At baseline, there were 1251 residents on the household occupancy lists in the population census (March-September 2010), of which 1013 (81%) consented to participate. Most participants (n=910, 90%) were seen over a four month period (April-July)……………… At month 12, there were 1110 residents on the household occupancy lists in the second population census (April-October 2011), of whom 1060 (95%) participated (~150 per month). …’* |
| Outcome data | 15* | *Cohort study*—Report numbers of outcome events or summary measures over time, p. 7,  Table 2. Participant details for the population census at month 0 and 12.  p. 8, Figure 1. Scabies prevalence at population censuses (2010 & 2011) and month 6 & 18 surveys.  p.9, **Figure 2. Flowchart of visits to participants in 13 priority houses.** |
|  |  | *Cross-sectional study—*Report numbers of outcome events or summary measures, p. 9, *‘****Month 6 and 18:*** *Scabies treatment failures and acquisition were low throughout the study period (Table S1 & S2). The treatment failure rate was 6% (2/35) at month 6 and 5% (5/91) at month 18. The acquisition rate was 1% (4/352) at month 6 and 2% (6/276) at month 18. The median time between participant visits from baseline to month 6 was five months (IQR 5-8 months) and from month 12 to 18, eight months (IQR 6-11).’* |
| Main results | 16 | (*a*) Give unadjusted estimates and, if applicable, confounder-adjusted estimates and their precision (eg, 95% confidence interval). Make clear which confounders were adjusted for and why they were included. N/A population census. |
|  |  | (*b*) Report category boundaries when continuous variables were categorized, Figures 1-4. |
|  |  | (*c*) If relevant, consider translating estimates of relative risk into absolute risk for a meaningful time period, p. 7*, ‘At the month 6 survey, prevalence was 1% but increased to 9% at month 12 (5% absolute increase for the baseline cohort).’* |
| Other analyses | 17 | Report other analyses done—eg analyses of subgroups and interactions, and sensitivity analyses, p. 8 & Figure 2. |
| Discussion p. 9-11 | | |
| Key results | 18 | Summarise key results with reference to study objectives, p.9, *‘In our study, MDA incorporating ivermectin had a demonstrable but relatively short-term impact on scabies prevalence. In the six-months following each MDA, both the low overall prevalence (1-3%) and the low acquisition rates (1-2%) suggest that transmission was substantially reduced. However, the rapid rise in prevalence at month 12 highlights that an MDA program, where utilised, needs to be incorporated with a multi-faceted control program and ongoing surveillance in the community.’* |
| Limitations | 19 | Discuss limitations of the study, taking into account sources of potential bias or imprecision. Discuss both direction and magnitude of any potential bias, p.10, ‘*The high proportion of new entries to the cohort at the month 12 census (36%) coincided with a large funeral that was attended by visitors from other communities who were camping in tents in the house yards of relatives. At this time, many local residents were also displaced from their homes into tents or other people’s homes as their houses were being refurbished or demolished and rebuilt, as part of a government initiative to address housing shortages in Aboriginal communities…..…*  *It was also apparent to our community-based research team, that the relationship built over the course of the team’s work meant that by month 12 it was relatively commonplace for households to seek out the research team to assist in making their homes scabies free, and to send family members who had not been present on the day the family were seen to the research office for screening and treatment. We acknowledge that this may have introduced a screening bias in the latter part of the study but the increased scabies prevalence at month 12 amongst those who had been seen at baseline indicates that the increase in prevalence was not an artefact of care-seeking behaviour.’* |
| Interpretation | 20 | Give a cautious overall interpretation of results considering objectives, limitations, multiplicity of analyses, results from similar studies, and other relevant evidence, p. 11*, ‘The rise in scabies prevalence at month 12 coincided with: a cluster of cases epidemiologically-linked to an individual with presumptive crusted scabies, a high prevalence amongst new entries to the cohort (an indicator of the impact of high population mobility), and an increased prevalence amongst members of the baseline cohort who did not have a known exposure to the suspected crusted scabies case (4% to 7%). This demonstrated how readily scabies prevalence can increase.’* |
| Generalisability | 21 | Discuss the generalisability (external validity) of the study results, p. 11, *‘Our study provides evidence that ivermectin based MDAs can have a role in reducing scabies prevalence but also highlights that maintaining a reduction requires ongoing surveillance, diagnosis and chronic case management of individuals with crusted scabies, and ongoing engagement with community members that has a particular focus on households and close contacts. Due to the customary movements of Aboriginal people, regional approaches to decrease re-introduction of scabies from neighbouring communities needs to be considered.’* |
| Other information | | |
| Funding | 22 | Give the source of funding and the role of the funders for the present study and, if applicable, for the original study on which the present article is based, p. 11, ‘*This work was supported by National Health and Medical Research Council (GTN0605804); Cooperative Research Centre for Aboriginal Health (HS331) and Northern Territory Research Innovation Board and Fund (Grant round 6-2008).’* |

*Give information separately for cases and controls in case-control studies and, if applicable, for exposed and unexposed groups in cohort and cross-sectional studies.

**Note:** An Explanation and Elaboration article discusses each checklist item and gives methodological background and published examples of transparent reporting. The STROBE checklist is best used in conjunction with this article (freely available on the Web sites of PLoS Medicine at http://www.plosmedicine.org/, Annals of Internal Medicine at http://www.annals.org/, and Epidemiology at http://www.epidem.com/). Information on the STROBE Initiative is available at www.strobe-statement.org.
